# Temporal trends in diagnoses and mental healthcare utilisation in child and adolescent psychiatry from 2013 to 2023

**DOI:** 10.1007/s00787-026-03008-4

**Published:** 2026-03-16

**Authors:** Kirstie O’Hare, Ulla Lång, Valentina Kieseppä, Hugh Ramsay, Maija Lindgren, Atte Kyrölä, David Gyllenberg, Ian Kelleher

**Affiliations:** 1 Institute for Neuroscience and Cardiovascular Research, University of Edinburgh, Edinburgh, UK; 2 Discipline of Psychiatry and Mental Health, University of New South Wales, Sydney, Australia; 3 Faculty of Medicine, University of Oulu, Oulu, Finland; 4 Finnish Institute for Health and Welfare, Helsinki, Finland; 5 Department of Adolescent Psychiatry, Helsinki University Hospital, Helsinki, Finland; 6 Department of Child Psychiatry and Invest Flagship, University of Turku, Turku, Finland; 7 School of Medicine, University College Dublin, Dublin, Ireland; 8 St John of God Hospitaller Services Group, Stillorgan, Dublin, Ireland

**Keywords:** Service utilisation, Child and adolescent mental health services, Time trends, Administrative data

## Abstract

Youth mental health has declined over the past two decades, driving increased demand for child and adolescent psychiatry services. Little is known, however, about how diagnoses and related resource allocation is changing. Our aim was to analyse trends in child and adolescent psychiatry diagnoses and service use over time. We examined administrative health record data for all child and adolescent psychiatry service appointments between 2013 and 2023 for children (aged 0–12 years) and adolescents (aged 13–17 years) living in Finland. Stratifying by diagnosis, annual rates were calculated for inpatient and outpatient service use. Negative binomial regression was used to assess trends over time across diagnostic categories. The number of individuals attending psychiatric services rose by 137.5% in children and 112.4% in adolescents. Total number of appointments increased by 62.3% in children and 50.3% in adolescents. The mean number of appointments per individual declined from 7.8 to 5.3 for children and 7.9 to 5.6 for adolescents. Most diagnostic categories had increased presentations, with the largest absolute increases for attention deficit hyperactivity disorder (ADHD) in children (+ 59,138 appointments, average annual percentage change [AAPC] 15.28%) depressive disorders in adolescents (+ 30,213 appointments, AAPC 5.16%), and anxiety disorders in adolescents (+ 28,027 appointments, AAPC 6.04%). Number of appointments for people with schizophrenia-spectrum and bipolar disorders declined despite stable case numbers. Overall, CAMHS attendance has dramatically increased with substantial variation by diagnosis. Declining clinical time allocated to individuals with schizophrenia-spectrum and bipolar disorders raises concerns about equitable service access for young people with severe mental illnesses.

**Supplementary Information:**

The online version contains supplementary material available at 10.1007/s00787-026-03008-4.

## Introduction

The mental health of young people has been declining over the past two decades. Many studies in both general population and clinical samples suggest an increased prevalence of mental ill health in young people [1, 3, 5, 7, 11, 19, 21, 35, 36, 40, 45]. These findings have led experts to recognise a youth mental health crisis as a major public health concern [32].

Temporal trends in the use of child and adolescent psychiatry services, however, including outpatient appointments and inpatient beds, as well as the relationship between service use and different mental disorder diagnoses, remain unclear. Recent reports have established a large increase in the number of children and adolescents presenting to mental health services [2, 20, 37]. These reports, however, have not examined how clinical time and inpatient resources are allocated across diagnostic categories or how this has changed over time. Quantifying changes over time in child and adolescent psychiatry service use and the relationship with mental disorder diagnoses represents critical healthcare intelligence data which should inform service planning and resource allocation.

The aim of this study was to describe temporal trends in child and adolescent psychiatry service use and related mental disorder diagnoses for the whole population age < 18 years living in Finland between 2013 and 2023. We examined changes for both the number of individuals coming to services and the total numbers of outpatient appointments and inpatient bed days across diagnostic categories.

## Methods

### Study design and participants

In Finland, universal healthcare is provided to residents through a tax-funded system. The Finnish Care Register for Health Care captures detailed data on specialist health care contacts, including information on the specialty of the treating physician (e.g. psychiatry). Using this register, we identified all individuals who had utilised specialist child and adolescent psychiatry services between 2013 and 2023. Population data were also obtained from Statistics Finland’s database to define annual population denominators for rate calculations [43]. There was little change in the child or adolescent populations in Finland over the study period (Supplementary Table 1).

### Mental health service contacts

Child and adolescent psychiatry services in Finland are specialist public mental health services that provide free-at-the-point-of-access care to young people referred from primary care, including inpatient, outpatient, home-based and early intervention psychosis services for under 18-year-olds. They do not include mental health services provided within a primary care or school setting. Child and adolescent psychiatry contacts were identified through the Care Register for Health Care and grouped into diagnostic groups defined using International Statistical Classification of Diseases and Related Health Problems, 10th Revision (ICD-10) codes, which was the coding system used in Finland during the study period. We examined inpatient admissions and outpatient contacts, categorising each based on which disorder was coded as the primary diagnosis associated with the service contact. Outpatient contacts included remote contacts from 2019 onwards, following the introduction of this category into the patient records system.

Diagnoses were classified into the following categories: (1) Substance use disorders (F10-F19), (2) Schizophrenia, schizotypal disorders, and delusional disorders (F20-F29), (3) Mania or bipolar disorder (F30-F31), (4) Depressive disorders (F32-F33), (5) Anxiety disorders (F40-F41), (6) Obsessive compulsive disorder (F42), (7) Eating disorders (F50), (8) Personality disorders (F60-F69), (9) Autism spectrum disorders (F84.0-F84.1, F84.5), (10) Attention-deficit hyperactivity disorder (F90), and (11) Conduct or oppositional defiant disorders (F91-F92). Categories were not mutually exclusive (i.e., one individual could have diagnoses in more than one different disorder category). Total outpatient and inpatient mental health service use numbers included service use for any diagnostic category from F00-F99, as well as mental health service use where the diagnosis was coded as ‘other’ or missing.

### Analysis

We first conducted descriptive analyses, presenting annual rate per 100 population for (1) number of inpatient bed days, (2) number of individuals with an inpatient admission, (3) number of outpatient appointments, and (4) number of individuals with an outpatient appointment, for each diagnostic category, and for the total amount of service use across all diagnostic categories. We presented these rates stratified by whether the visit occurred in childhood (aged 0–12 years [inclusive]) or adolescence (aged 13 to 17 years [inclusive]). Negative binomial regression models were fitted to data stratified by age group and diagnostic category. Calendar year (as a continuous variable) was used as the predictor variable, and the outcome variables were: number of inpatient bed days, number of individuals with an inpatient admission, number of outpatient appointments and number of individuals with an outpatient appointment. To account for changes in the underlying population over time, the log of the number of individuals at risk (i.e., all 0–12 year olds or all 13–17 year olds in Finland) was included as an offset term in the model. Coefficients were exponentiated and interpreted as the average annual percentage change in the outcome.

Data were also visualised with tree maps using the R package ‘treemap’ [46]. In these figures, each diagnostic category is represented by a rectangle. The size of each rectangle reflects the relative proportion of either inpatient bed days or outpatient appointments that were attributed to each diagnostic category. We generated these figures for both the earliest year of available data (2013), and the latest year (2023), to provide a visual representation of how service use has changed over time in child and adolescent psychiatry services.

Analyses were conducted using R version 4.4.1.

### Results

#### Outpatient setting

**Fig. 1:**
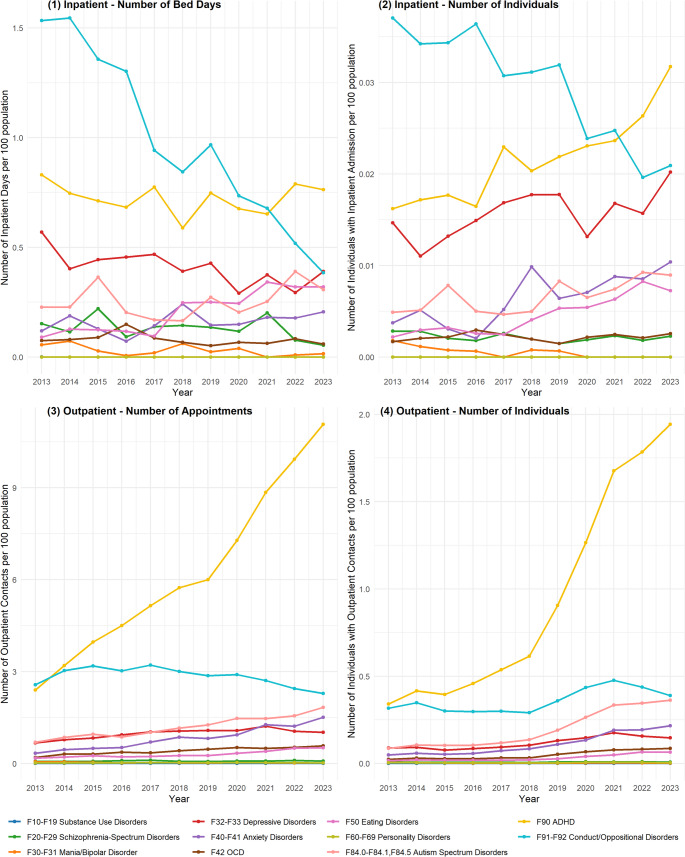
Inpatient admissions and outpatient appointments in child psychiatry in Finland, 2013–2023

*Child Psychiatry.* Overall, the number of individuals with outpatient appointments for child psychiatry increased over time, from 21,191 children in 2013 to 50,329 in 2023 (a 137.5% increase; Table 1). The number of outpatient appointments also increased over time, from 165,214 appointments in 2013 to 268,215 appointments in 2023 (a 62.3% increase), and the mean number of appointments reduced from 7.8 appointments per patient in 2013 to 5.3 in 2023. In 2013, conduct and oppositional disorders were the most common diagnoses associated with outpatient psychiatry appointments for children. From 2014 to 2023, however, ADHD became the most common diagnosis associated with outpatient appointments (Figs. 1 and 3).

**Table 1:** Change in total amounts of inpatient and outpatient psychiatry service use for children and adolescents in Finland, 2013–2023

	Inpatient setting															
	Child Psychiatry								Adolescent Psychiatry							
**Year**	Total bed days	% change from previous year		Total indivi-duals	% change from previous year	Mean bed days per admitted patient		% change from previous year	Total bed days	% change from previous year		Total indivi-duals		% change from previous year	Mean bed days per admitted patient	% change from previous year
**2013**	47,487			1,069		44.4			94,973			2,384			39.8	
**2014**	44,439	−6.4%		1,059	−0.9%	42.0		−5.5%	85,025	−10.5%		2,315		−2.9%	36.7	−7.8%
**2015**	44,623	0.4%		1,139	7.6%	39.2		−6.6%	78,834	−7.3%		2,263		−2.2%	34.8	−5.2%
**2016**	39,764	−10.9%		1,096	−3.8%	36.3		−7.4%	78,978	0.2%		2,508		10.8%	31.5	−9.6%
**2017**	38,010	−4.4%		1,163	6.1%	32.7		−9.9%	86,892	10.0%		3,006		19.9%	28.9	−8.2%
**2018**	34,959	−8.0%		1,139	−2.1%	30.7		−6.1%	76,408	−12.1%		3,035		1.0%	25.2	−12.9%
**2019**	34,488	−1.3%		1,140	0.1%	30.3		−1.4%	79,394	3.9%		3,201		5.5%	24.8	−1.5%
**2020**	30,496	−11.6%		1,039	−8.9%	29.4		−3.0%	73,500	−7.4%		3,354		4.8%	21.9	−11.6%
**2021**	32,251	5.8%		1,103	6.2%	29.2		−0.4%	76,875	4.6%		3,605		7.5%	21.3	−2.7%
**2022**	29,893	−7.3%		1,042	−5.5%	28.7		−1.9%	77,958	1.4%		3,447		−4.4%	22.6	6.1%
**2023**	29,915	0.1%		1,164	11.7%	25.7		−10.4%	83,885	7.6%		3,536		2.6%	23.7	4.9%
**% change from 2013 to 2023**		−37.0%			8.9%			−42.1%		−11.7%				48.3%		−40.5%
	Outpatient setting															
	Child Psychiatry								Adolescent Psychiatry							
**Year**	Total appoint-ments	% change from previous year	Total indivi-duals		% change from previous year		Mean number of appointments per patient	% change from previous year	Total appoint-ments		% change from previous year		Total indivi-duals	% change from previous year	Mean number of appointments per patient	% change from previous year
**2013**	165,214		21,191				7.8		234,298				29,693		7.9	
**2014**	177,880	7.7%	23,211		9.5%		7.7	−1.7%	246,360		5.1%		31,863	7.3%	7.7	−2.0%
**2015**	204,902	15.2%	22,404		−3.5%		9.1	19.3%	263,486		7.0%		29,334	−7.9%	9.0	16.2%
**2016**	220,920	7.8%	25,423		13.5%		8.7	−5.0%	275,424		4.5%		31,929	8.8%	8.6	−4.0%
**2017**	237,628	7.6%	27,950		9.9%		8.5	−2.2%	317,309		15.2%		36,228	13.5%	8.8	1.5%
**2018**	246,420	3.7%	29,720		6.3%		8.3	−2.5%	322,771		1.7%		37,670	4.0%	8.6	−2.2%
**2019**	239,857	−2.7%	36,110		21.5%		6.6	−19.9%	315,609		−2.2%		42,676	13.3%	7.4	−13.7%
**2020**	242,332	1.0%	41,821		15.8%		5.8	−12.8%	331,311		5.0%		53,081	24.4%	6.2	−15.6%
**2021**	255,874	5.6%	48,761		16.6%		5.2	−9.4%	364,043		9.9%		61,333	15.5%	5.9	−4.9%
**2022**	251,736	−1.6%	47,227		−3.1%		5.3	1.6%	345,906		−5.0%		61,353	0.0%	5.6	−5.0%
**2023**	268,215	6.5%	50,329		6.6%		5.3	0.0%	352,047		1.8%		63,079	2.8%	5.6	−1.0%
**% change from 2013 to 2023**		62.3%			137.5%			−31.6%			50.3%			112.4%		−29.3%

There were increases both in the number of appointments and in the number of individuals with outpatient contacts for most diagnostic categories: depressive disorders, anxiety disorders, OCD, eating disorders, autism spectrum disorders, and ADHD. The largest increase was seen for ADHD, which increased 317.0% from 18,655 appointments in 2013 to 77,793 in 2023 (for 2,647 and 13,656 children respectively [415.9% increase]; Supplementary Tables 1 and 2).

There was no change in the number of children diagnosed with mania and bipolar disorder but there was a significant decrease in the numbers of outpatient appointments for children with these diagnoses (Table 2). The same was true for conduct and oppositional disorders, with no change in the number of children with these diagnoses but a significant decrease in the number of appointments for children with these diagnoses (Table 2).

**Table 2:** Negative binomial regression of relationship between calendar year and numbers of inpatient and outpatient psychiatry service use, stratified by selected diagnostic groups, from 2013 to 2023

Child Psychiatry	Average annual percentage change (95% CI)		*p*	Absolute Change†		Average annual percentage change (95% CI)	*p*	Absolute Change†
	Inpatient Setting							
	Number of Bed Days					Number of Individuals		
Substance Use Disorders	-		-	-		-	-	
Schizophrenia-Spectrum Disorders	−4.97% (−11.24%, 1.70%)		0.118	−808		−2.91% (−7.36%, 1.71%)	0.215	−6
Mania/Bipolar Disorder	−14.23% (−29.74%, 4.38%)		0.112	−325		−29.20% (−38.18%, −20.18%)	< 0.001***	−9††
Depressive Disorders	−4.07% (−6.32%, −1.77%)		0.001**	−1,684		3.24% (0.87%, 5.67%)	0.007**	+ 28
Anxiety Disorders	4.09% (−0.93%, 9.35%)		0.115	+ 524		11.69% (5.80%, 17.97%)	< 0.001***	+ 44
OCD	−4.05% (−8.49%, 0.62%)		0.081	−162		1.22% (−3.37%, 6.02%)	0.609	+ 5
Eating Disorders	15.02% (10.60%, 19.62%)		< 0.001***	+ 1,543		14.30% (10.53%, 18.25%)	< 0.001***	+ 34
Personality Disorders	-		-	-		-	-	-
Autism Spectrum Disorders	2.72% (−1.88%, 7.55%)		0.273	+ 396		5.87% (2.41%, 9.47%)	0.001**	+ 25
ADHD	−0.46% (−2.11%, 1.22%)		0.600	−1,092		6.28% (4.71%, 7.88%)	< 0.001***	+ 97
Conduct/Oppositional Disorders	−12.27% (−14.11%, −10.39%)		< 0.001***	−9,223		−5.83% (−7.30%, −4.33%)	< 0.001***	−141
	Outpatient Setting							
	Number of Appointments					Number of Individuals		
Substance Use Disorders	−0.36% (−10.34%, 10.70%)		0.945	< 5		−1.14% (−13.49%, 12.51%)	0.864	< 5
Schizophrenia-Spectrum Disorders	1.49% (−1.43%, 4.51%)		0.314	+ 17		2.92% (0.01%, 5.91%)	0.050*	−11
Mania/Bipolar Disorder	−10.41% (−14.30%, −6.37%)		< 0.001***	−324		−6.04% (−12.22%, 0.48%)	0.091	−33
Depressive Disorders	4.43% (2.55%, 6.35%)		< 0.001***	+ 1,924		8.12% (5.67%, 10.62%)	< 0.001***	+ 344
Anxiety Disorders	15.26% (13.57%, 16.96%)		< 0.001***	+ 7,963		17.94% (15.39%, 20.56%)	< 0.001***	+ 1,137
OCD	9.22% (7.22%, 11.25%)		< 0.001***	+ 2,494		15.68% (12.50%, 18.96%)	< 0.001***	+ 428
Eating Disorders	10.73% (8.48%, 13.03%)		< 0.001***	+ 2,261		16.09% (12.37%, 19.94%)	< 0.001***	+ 333
Personality Disorders	4.27% (−0.26%, 8.99%)		0.048	−13		4.38% (0.13%, 8.82%)	0.044*	−7
Autism Spectrum Disorders	9.31% (8.18%, 10.45%)		< 0.001***	+ 7,450		17.48% (14.49%, 20.56%)	< 0.001***	+ 1,875
ADHD	15.28% (13.96%, 16.61%)		< 0.001***	+ 59,138		21.43% (18.44%, 24.50%)	< 0.001***	+ 11,009
Conduct/Oppositional Disorders	−1.96% (−3.55%, −0.35%)		0.014**	−3,896		3.98% (1.73%, 6.28%)	0.001**	+ 274
Adolescent Psychiatry	Average annual percentage change (95% CI)		p	Absolute Change†		Average annual percentage change (95% CI)	p	Absolute Change†
	Inpatient Setting							
	Number of Bed Days					Number of Individuals		
Substance Use Disorders	−6.44% (−11.39%, −1.25%)		0.012*	−402		7.39% (0.75%, 14.46%)	0.018*	+ 17
Schizophrenia-Spectrum Disorders	−7.39% (−9.79%, −4.91%)		< 0.001***	−6164		−3.23% (−4.63%, −1.82%)	< 0.001***	−27
Mania/Bipolar Disorder	−10.41% (−14.33%, −6.30%)		< 0.001***	−2085		−3.07% (−5.72%, −0.35%)	0.027*	−30
Depressive Disorders	−1.45% (−2.70%, −0.18%)		0.028*	−3872		5.80% (4.08%, 7.54%)	< 0.001***	+ 462
Anxiety Disorders	−1.28% (−2.65%, 0.11%)		0.065	−15		6.87% (5.05%, 8.73%)	< 0.001***	+ 215
OCD	−4.97% (−9.06%, −0.71%)		0.029*	−1246		5.26% (1.77%, 8.89%)	0.003**	+ 16
Eating Disorders	4.57% (2.43%, 6.74%)		< 0.001***	+ 6,015		9.17% (6.79%, 11.60%)	< 0.001***	+ 145
Personality Disorders	5.67% (−8.11%, 21.28%)		0.344	−19		10.12% (2.21%, 18.78%)	0.007**	+ 13
Autism Spectrum Disorders	3.60% (−1.80%, 9.30%)		0.164	+ 143		7.88% (4.93%, 10.94%)	< 0.001***	+ 41
ADHD	−2.61% (−6.78%, 1.74%)		0.226	−788		7.75% (4.40%, 11.22%)	< 0.001***	+ 51
Conduct/Oppositional Disorders	−10.05% (−13.53%, −6.44%)		< 0.001***	−5193		−5.07% (−6.86%, −3.25%)	< 0.001***	−115
	Outpatient Setting							
	Number of Appointments					Number of Individuals		
Substance Use Disorders	−1.46% (−5.89%, 3.19%)		0.515	+ 174		3.75% (0.17%, 7.46%)	0.035*	+ 59
Schizophrenia-Spectrum Disorders	−3.96% (−5.75%, −2.14%)		< 0.001***	−836		1.56% (−0.36%, 3.51%)	0.118	+ 15
Mania/Bipolar Disorder	−0.33% (−2.44%, 1.82%)		0.760	+ 56		4.73% (2.31%, 7.20%)	< 0.001***	+ 80
Depressive Disorders	5.16% (2.42%, 7.97%)		< 0.001***	+ 30,213		10.66% (8.61%, 12.75%)	< 0.001***	+ 5,688
Anxiety Disorders	6.04% (3.80%, 8.33%)		< 0.001***	+ 28,027		12.45% (10.51%, 14.43%)	< 0.001***	+ 6,197
OCD	6.90% (5.66%, 8.16%)		< 0.001***	+ 5,256		14.27% (11.50%, 17.11%)	< 0.001***	+ 1,120
Eating Disorders	6.13% (4.50%, 7.78%)		< 0.001***	+ 9,709		11.38% (7.91%, 14.95%)	< 0.001***	+ 1,503
Personality Disorders	2.43% (−2.15%, 7.23%)		0.258	+ 272		10.22% (5.10%, 15.60%)	< 0.001***	+ 176
Autism Spectrum Disorders	6.27% (5.19%, 7.36%)		< 0.001***	+ 5,506		14.82% (12.36%, 17.34%)	< 0.001***	+ 1,959
ADHD	13.82% (13.09%, 14.55%)		< 0.001***	+ 27,025		19.18% (16.20%, 22.22%)	< 0.001***	+ 6,759
Conduct/Oppositional Disorders	−7.31% (−9.26%, −5.31%)		< 0.001***	−6,548		−1.74% (−3.57%, 0.13%)	0.066	−569

*ADHD* attention deficit hyperactivity disorder, *OCD* obsessive compulsive disorder

*p < 0.05, **p < 0.01, ***p < 0.001

†Absolute difference between number in 2013 and number in 2023

††Difference shown is between 2013 and 2019, as the 2023 data was withheld to meet statistical disclosure requirements

**Fig. 2:**
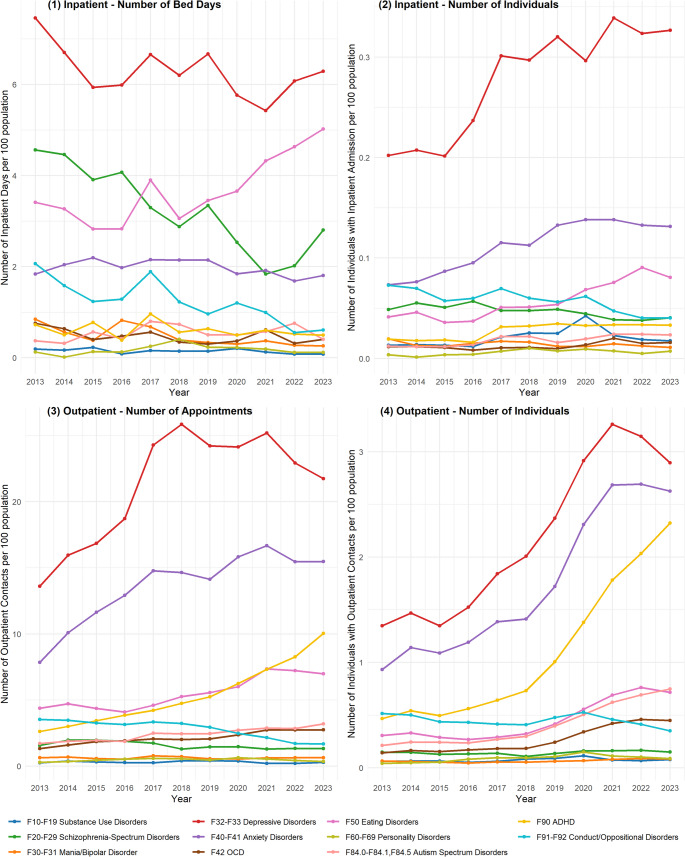
Inpatient admissions and outpatient appointments in adolescent psychiatry in Finland, 2013–2023. ADHD, attention deficit hyperactivity disorder; OCD, obsessive compulsive disorder

*Adolescent Psychiatry.* Overall, the number of individuals with outpatient appointments in adolescent psychiatry increased from 29,693 adolescents in 2013 to 63,079 adolescents in 2023 (a 112.4% increase; Table 1). The number of outpatient adolescent psychiatry appointments also increased, from 234,298 appointments in 2013 to 352,047 in 2023 (a 50.3% increase), and the mean number of appointments reduced from 7.9 appointments per patient in 2013 to 5.6 in 2023. Depressive disorders, followed by anxiety disorders, were the most prevalent diagnoses associated with adolescent outpatient psychiatry appointments for the entire time period from 2013 to 2023 (Figs. 2 and 4). There were increases both in the number of appointments and in the number of adolescents with outpatient contacts for most diagnostic categories: depressive disorders, anxiety disorders, OCD, eating disorders, autism spectrum disorders, and ADHD (Table 2). The largest increase was seen for depressive disorders, which increased 62.1% from 48,681 appointments in 2013, to 78,894 appointments in 2023 (for 4,819 and 10,507 adolescents respectively [118% increase]; Supplementary Tables 3 and 4).

**Fig. 3:**
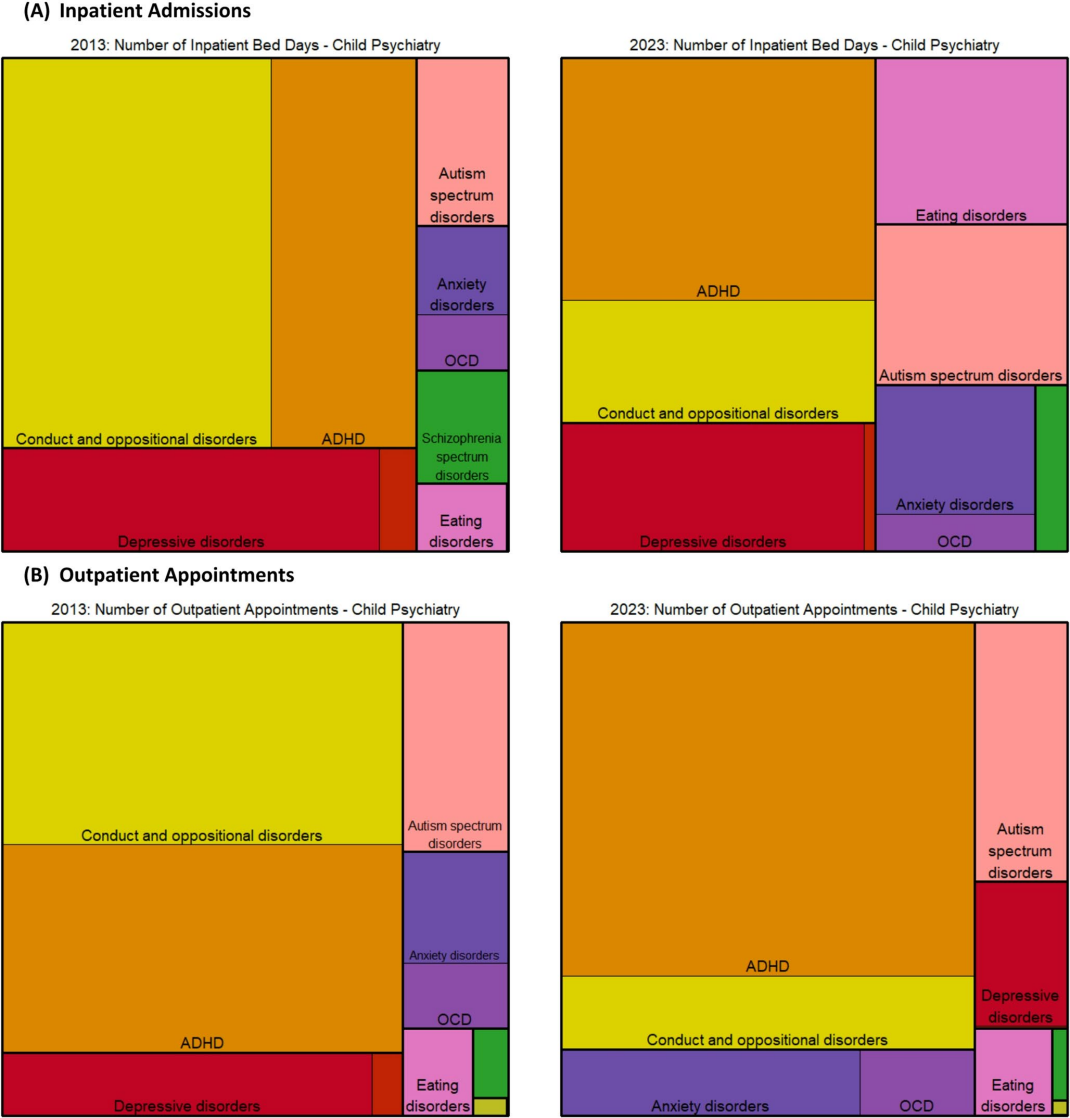
Proportions of inpatient admissions and outpatient appointments associated with different diagnostic group in child psychiatry in Finland, in 2013 and 2023. ADHD, attention deficit hyperactivity disorder; OCD, obsessive compulsive disorder

There was no change in the number of adolescents diagnosed with schizophrenia-spectrum disorders but there was a significant decrease in the number of outpatient appointments for adolescents with these diagnoses (Table 2). The same was true for conduct and oppositional disorders, with no change in the number of adolescents with these diagnoses but a significant decrease in the number of appointments for adolescents with these diagnoses (Table 2). There was an increase in the number of adolescents diagnosed with personality disorders but there was no corresponding change in the number of appointments for adolescents with personality disorders. (Figure 3)

#### Inpatient Setting

**Fig. 4:**
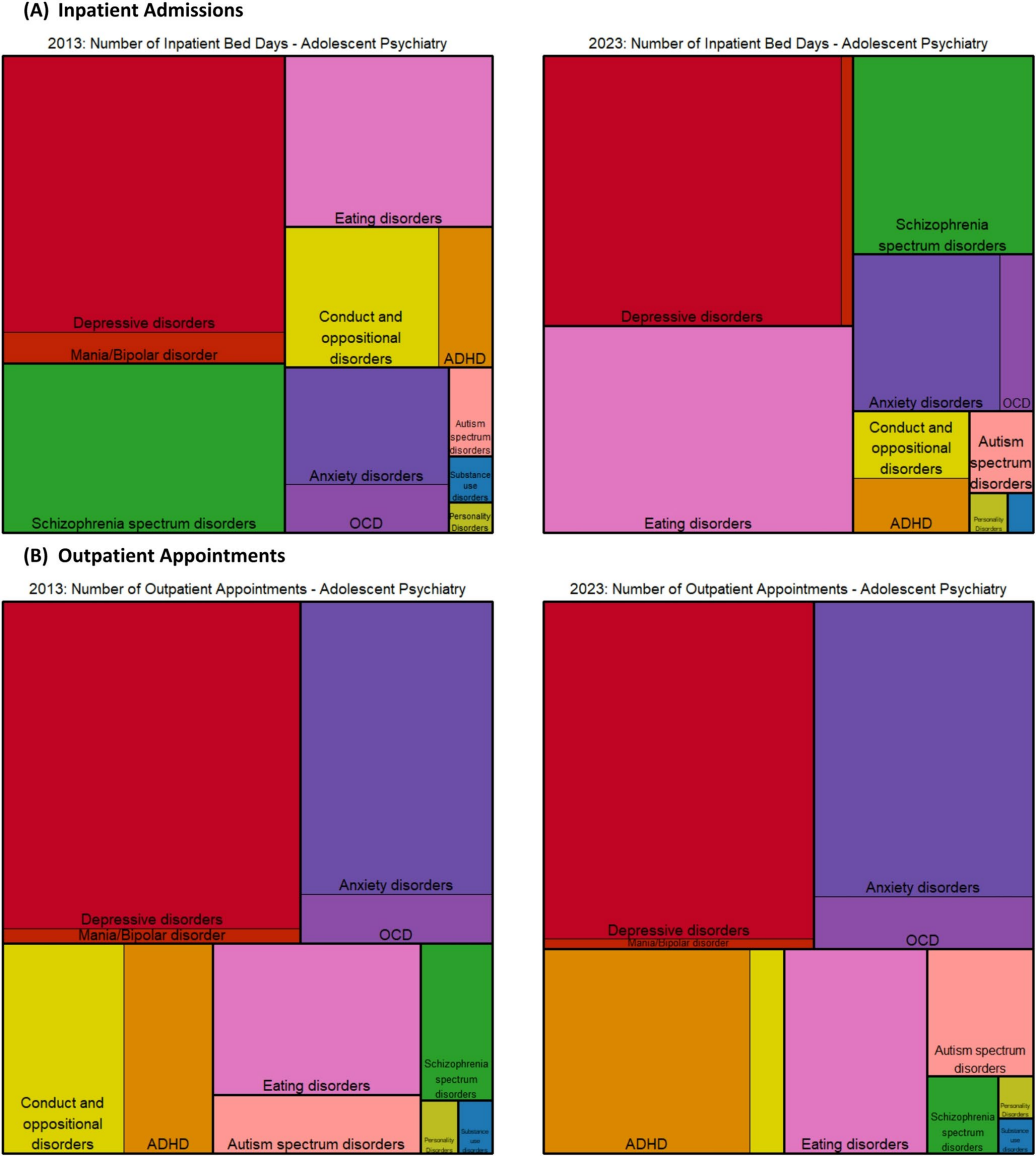
Proportions of inpatient admissions and outpatient appointments associated with different diagnostic groups in adolescent psychiatry in Finland, in 2013. and 2023. ADHD, attention deficit hyperactivity disorder; OCD, obsessive compulsive disorder

*Child Psychiatry.* Overall, the number of individuals with inpatient admissions increased over time, from 1,069 children in 2013 to 1,164 children in 2023 (an 8.9% increase; Table 1). The overall mean length of inpatient admission was 34.9 days (SD = 17.3 days), declining from 44.4 days in 2013 to 25.7 days in 2023. In contrast, the total number of inpatient bed days for child psychiatry decreased over time, from 47,487 total days in 2013 to 29,915 days in 2023 (a 37% decrease). The diagnostic category associated with both the highest number of children admitted and total numbers of inpatient bed days for children was conduct or oppositional disorders from 2013 to 2021, and then ADHD from 2022 to 2023 (Figs. 1 and 3). For mania and bipolar disorders, and conduct and oppositional disorders, both the number of children admitted and the numbers of inpatient bed days significantly decreased from 2013 to 2023 (Table 2). For depressive disorders, the number of inpatient bed days reduced significantly over time, but there was no change in the number of children admitted. For anxiety disorders, autism spectrum disorders, and ADHD, the number of children admitted increased significantly from 2013 to 2023, but there was no change in the number of associated bed days. The only diagnostic category for which both number of children and number of bed days increased significantly over time was eating disorders, which increased 219.5% from 703 bed days in 2013 to 2,246 bed days in 2023 (for 17 and 51 children respectively [200% increase]; Supplementary Tables 1 and 2). (Figure 4)

*Adolescent Psychiatry.* Overall, the total number of adolescents with inpatient admissions increased from 2,384 adolescents in 2013 to 3,536 adolescents in 2023 (a 48.3% increase; Table 1). The overall mean length of inpatient admission was 30.1 days (SD = 19.2 days), declining from 39.8 days in 2013 to 23.7 days in 2023. However, the total number of inpatient bed days for adolescent psychiatry decreased over time, from 94,973 total days in 2013 down to 83,885 days in 2023 (a 11.7% decrease). The diagnostic group associated with the highest number of both adolescents with inpatient admissions and bed days for adolescent psychiatry was depressive disorders (Figs. 2 and 4). For both conduct and oppositional disorders, and schizophrenia-spectrum disorders, the number of both adolescents admitted and inpatient bed days decreased – for schizophrenia-spectrum disorders this decreased 37.7% from 16,333 bed days in 2013, to 10,169 bed days in 2023 (for 174 and 147 adolescents respectively [15.5% decrease]; Supplementary Tables 3 and 4). For depressive disorders, anxiety disorders, OCD, personality disorders, autism spectrum disorders, and ADHD the number of adolescents admitted increased while there was no significant change to the number of inpatient bed days over time. For mania and bipolar disorders there was no significant change in the number of adolescents admitted but there was a significant decrease in the number of inpatient bed days for these diagnoses. For eating disorders, both the number of adolescents and number of bed days in the inpatient setting increased significantly over time.

## Discussion

The number of young people attending outpatient mental health services more than doubled over the previous decade. There was a 138% increase in the number of children attending psychiatry services, and a 112% increase in the number of adolescents attending psychiatry services. The total number of outpatient appointments also increased over the same period, by 62% for children and 50% for adolescents.

Overall, there was a large increase in specialist mental health service use for children and adolescents across this time period. This is consistent with evidence reported globally that has indicated both a rise in mental health problems reported by young people [5, 9, 36, 40], and a corresponding rise in child and adolescent mental health service use [2, 20, 37]. We observed both a large increase in the number of individuals attending child and adolescent psychiatry services and in the total number of psychiatric appointments. The mean number of outpatient appointments decreased from ~8 to ~5 appointments per patient for both children and adolescents, highlighting that the increase in individuals attending child and adolescent psychiatry has resulted in a decrease in the amount of care each individual receives. While the number of individuals admitted to inpatient psychiatry wards also increased, the overall number of inpatient bed days decreased over time, meaning admissions have become shorter over time, with the mean length of stay almost halving - declining from 44 days to 26 days for children and from 40 days to 24 days for adolescents.

The diagnosis with the largest increase in service use was ADHD, particularly for children. There were also large increases in presentations for eating disorders, depressive disorders, and anxiety disorders, particularly for adolescents. In contrast, while there was no or little change in the number of children and adolescents being diagnosed with schizophrenia-spectrum and bipolar disorders, the amount of clinical time given to young people with these conditions, both in terms of number of inpatient bed days and outpatient appointments, has decreased over time.

In terms of inpatient treatment, there was an increase (8.9%) in the number of children receiving an inpatient mental health admission but, despite this, the total number of inpatient bed days decreased by 37%, from ~ 47,000 days in 2013 to ~ 30,000 days in 2023. In adolescents, there was a 48% increase in terms of the number of individuals receiving an inpatient admission but a 12% reduction in bed days, meaning that the average length of inpatient admissions reduced for this age group. The decreasing length of admissions may reflect a shift toward intensive outpatient care over inpatient admissions [13, 24], an increase in admissions due to crisis situations [30] and/or capacity pressures, with increased numbers of adolescents needing admissions necessitating shorter stays. Overall, the unequal growth between the volume of service utilization and the number of individuals accessing services highlights growing pressures on child and adolescent psychiatry services.

In terms of trends in specific diagnoses, the largest absolute increases in service use were seen for ADHD, consistent with other reports of an increasing trend of ADHD diagnoses globally [8, 10, 17, 33, 41, 47, 48]. This increase was particularly prominent for children, with the number of outpatient child psychiatry appointments for ADHD increasing from ~ 18,500 appointments in 2013 to over ~ 77,500 in 2023, a more than 4-fold increase. There are several reasons that may underlie the increase in ADHD diagnoses, for instance, some researchers have suggested that this increase reflects better identification and awareness of ADHD symptoms correcting previous underdiagnosis of this condition, particularly in girls [31, 49]. However, other researchers have suggested that overdiagnosis may be occurring [23], such as through broadening the definition of the disorder (i.e., diagnostic inflation) [6, 14].

In our data, we observed a decrease in service use for conduct disorders. Given that conduct disorders and ADHD are commonly comorbid and share overlapping impairments [15], this could suggest that there has been a shift in diagnostic or treatment focus from conduct disorders to ADHD. However, while the amount of service use for conduct disorders declined over time, the number of individuals with this diagnosis remained relatively stable. In contrast, the number of individuals with an ADHD diagnosis increased more than 5-fold, indicating that there are factors beyond a reclassification effect contributing to the rise in ADHD prevalence.

Other diagnostic groups that saw large increases include depressive disorders and anxiety disorders. Depressive disorders were the most common diagnoses associated with mental health service use among adolescents in both inpatient and outpatient settings. The amount of service use for depression has generally been increasing since 2013, though this peaked in 2021 with a small reduction in numbers since then. Anxiety disorders were the next most common diagnosis associated with adolescent mental health service use, and followed a similar increasing pattern to depressive disorders. The observed rise in anxiety and depressive disorders in adolescents is consistent with general population studies showing an increase in anxiety in young people [5, 9, 26, 28, 34], especially in adolescent girls. Notably, the rise in depressive and anxiety disorders occurred far in advance of the COVID-19 pandemic, and we did not observe any significant rise or fall in these numbers in 2020/2021 when the pandemic effects and restrictions were most significant. This suggests the post-pandemic increase in youth mental health problems is more likely a continuation of an existing trend, rather than a direct result of the pandemic, though our analyses were not designed to test causal hypotheses regarding COVID-19 effects.

We also observed a rising pattern of service use for eating disorders for both children and adolescents, though increases were much larger in adolescents compared to children. While much has been written about the increase in eating disorders in the context of the COVID-19 pandemic [12, 44], our longer-term data show that the increasing trajectory for eating disorders began before the pandemic, in 2017, accelerating further in 2018. This highlights the importance of investigating diagnostic trends over extended periods of time in order to see longer-term patterns in the data.

The number of outpatient appointments and inpatient bed days for both schizophrenia-spectrum disorders, and mania or bipolar disorders declined between 2013 and 2023, despite little to no change in the number of individuals coming to services with these disorders. While the decline in service use per person reflects a broader trend across all diagnostic categories, this is particularly concerning for those with severe mental illness because of the extent of the long-term disability [16, 29, 42] and premature mortality [4, 38] associated with these disorders, and there is evidence that they respond well to expert treatment, particularly if they are treated early [22, 39]. Undertreatment of these conditions in young people may therefore have long-term effects in increasing their likelihood of experiencing chronic and disabling mental ill-health into adulthood as well as other adverse health outcomes. This population faces a substantial advocacy gap: while organised advocacy has increased service access for individuals with neurodevelopmental disorders, the stigma and functional impairment often associated with severe mental illnesses, like schizophrenia, often prevent individuals and families from engaging in public-facing advocacy, leaving their needs systematically under-prioritised. These findings raise important concerns about equitable service access for individuals with severe mental illnesses. Given the potentially serious repercussions of undertreatment of these disorders, our findings point to the need for mental health services to explicitly protect capacity for treating severe mental illnesses. Reductions in duration of inpatient treatment could also be due to improvements in treatment; however, there were no major advances in schizophrenia treatment over the course of the study period [18, 27] making this explanation unlikely.

These findings have several implications for child and adolescent mental health services. The large increase in demand for child and adolescent psychiatry services over the past decade has resulted in a decline in the amount of service provision per person attending. This indicates that these specialist services are unable to continually expand to meet increasing demand without an impact on the quality of services provided. There is therefore a need to evaluate which young people can benefit most from treatments offered in child and adolescent psychiatry services, and whether there are young people attending these services who could benefit equally from other services.

The rapid change in individuals presenting to services also demonstrates the need for more research to evaluate the effectiveness of the interventions being provided. The evidence base for child and adolescent psychiatric interventions is largely based on trials conducted decades ago. Given the rapid change in the population attending these services, it is essential to evaluate the extent to which this evidence base still applies to modern (much-expanded) clinical populations. This highlights the need to invest in comprehensive research infrastructures within child and adolescent psychiatry services [25], including access to up-to-date mental health service use data, to ensure that mental health services are remaining responsive to the evolving needs of young people. Future research should also address the potential undertreatment of young people with severe mental illness, including understanding what types of treatments are being offered to these young people and the potential subsequent effects on mental health trajectories into adulthood.

Strengths of the study include the use of a whole population dataset and prospective administrative data which removes selection and recall bias. Further, the capacity to examine data on both the number of individuals and the volume of service use provides nuanced insights into patterns of mental health service use. Limitations of the study include that we did not have data on private mental health service contacts. However, as Finland has no private psychiatric hospitals, this limitation applies only to outpatient findings. Further, we relied on administrative health record data for information about mental health and neurodevelopmental disorder diagnoses. Diagnostic practices and documentation standards are not necessarily standardised across different sites and across years, potentially introducing some variability in accuracy. These data do, however, represent the real-world clinical diagnoses being assigned at a total population level. While our findings relate to Finland, an increase in child and adolescent psychiatry services presentations is a universal phenomenon for all countries and regions that are tracking these data. Our findings represent the most detailed analysis of these trends to date, including providing diagnostic data, which are missing from other studies. Our data, therefore, may be helpful to other areas experiencing large service use increases who do not have the detailed diagnostic and service use data that are essential for data-driven decision making.

In conclusion, these findings provide a comprehensive picture of temporal trends in mental health service use for children and adolescents over the past decade. The marked increase in service use, particularly for ADHD, depression, anxiety, and eating disorders, reflects a larger global trend of worsening mental health in young people identified at a general population level. At the same time, the declining amount of service provision per individual and the reduction in the amount of service use in particular for severe mental illnesses raises important concerns about ensuring that services are accessible to patients who are most at-risk of adverse long-term outcomes. These findings highlight the need for data-informed mental health services that can respond flexibly and promptly to changing patterns of need without compromising care for the most vulnerable.

## Supplementary Information

Below is the link to the electronic supplementary material.

Supplementary Material 1

## Data Availability

Access to the data was provided through the Finnish Institute for Health and Welfare, Finland. As data processors, rather than controllers, we do not have permission to provide public access to the data.
