# Supplementary material for: Temporal trends in diagnoses and mental healthcare utilisation in child and adolescent psychiatry from 2013 to 2023
Source: Eur Child Adolesc Psychiatry. 2026 Mar 16;35(6):1957–70. doi: 10.1007/s00787-026-03008-4 (PMC13337884; doi:10.1007/s00787-026-03008-4)
Supplement: Supplementary file 1 — Supplementary Material 1 [file 787_2026_3008_MOESM1_ESM.docx]

Supplementary Table 1. Total child and adolescent population numbers in Finland, 2013-2023

|  | Children  (0-12 years) | Adolescents (13-17 years) |
| --- | --- | --- |
| 2013 | 777362 | 357727 |
| 2014 | 780058 | 353104 |
| 2015 | 780322 | 348298 |
| 2016 | 777491 | 347569 |
| 2017 | 771241 | 345608 |
| 2018 | 761691 | 344063 |
| 2019 | 748934 | 345869 |
| 2020 | 737131 | 350919 |
| 2021 | 727224 | 357885 |
| 2022 | 713696 | 357470 |
| 2023 | 702874 | 362796 |

Supplementary Table 2. Numbers of inpatient psychiatry bed days for children and adolescents in Finland, 2013-2023

|  | **Number of Inpatient Days** | | | | | | | | | | |
| --- | --- | --- | --- | --- | --- | --- | --- | --- | --- | --- | --- |
| **Child Psychiatry** | **2013** | **2014** | **2015** | **2016** | **2017** | **2018** | **2019** | **2020** | **2021** | **2022** | **2023** |
| *Total inpatient bed days* | 47487 | 44439 | 44623 | 39764 | 38010 | 34959 | 34488 | 30496 | 32251 | 29893 | 29915 |
| F00-F09 Dementia and other organic brain syndromes | - | - | - | - | - | - | - | - | - | - | - |
| F10-F19 Substance Use Disorders | 8 | - | - | - | - | - | - | - | - | - | - |
| F20-F29 Schizophrenia-Spectrum Disorders | 1183 | 895 | 1718 | 717 | 1068 | 1102 | 1019 | 862 | 1465 | 550 | 375 |
| F30-F39 Mood Disorders | 5067 | 4038 | 4171 | 3996 | 5450 | 3852 | 3913 | 2957 | 3595 | 2722 | 3937 |
| *F30-F31 Mania/Bipolar Disorder* | 431 | 572 | 219 | 50 | 150 | 471 | 178 | 290 | 0 | 67 | 106 |
| *F32-F33 Depressive Disorders* | 4423 | 3142 | 3464 | 3541 | 3608 | 2979 | 3204 | 2141 | 2727 | 2098 | 2739 |
| F40-F48 Neurotic/Stress Disorders | 3477 | 3985 | 4052 | 3263 | 3874 | 4033 | 2768 | 3234 | 3757 | 3303 | 3264 |
| *F40-F41 Anxiety Disorders* | 925 | 1465 | 1007 | 557 | 1100 | 1838 | 1086 | 1102 | 1317 | 1273 | 1449 |
| *F42 OCD* | 580 | 623 | 700 | 1158 | 671 | 516 | 389 | 493 | 452 | 594 | 418 |
| F50-F59 Behavioural physiological/physical factors | 713 | 1067 | 974 | 927 | 751 | 1891 | 1877 | 1802 | 2489 | 2285 | 2253 |
| *F50 Eating Disorders* | 703 | 990 | 970 | 914 | 734 | 1885 | 1877 | 1802 | 2489 | 2285 | 2246 |
| F60-F69 Personality Disorders | - | - | - | - | - | - | - | - | - | - | - |
| *F60-F61 Adult personality/behavioral Disorders* | - | - | - | - | - | - | - | - | - | - | - |
| *F64 Gender Identity Disorders* | - | - | - | - | - | - | - | - | - | - | - |
| F70-F79 Intellectual Disabilities | 513 | 279 | 85 | 506 | 341 | 95 | 24 | 139 | 220 | 98 | 75 |
| F80-F89 Psychological Development Disorders | 6455 | 5720 | 7095 | 5249 | 5279 | 3707 | 4920 | 3551 | 3935 | 4329 | 4247 |
| *F84.0-F84.1,F84.5 Autism Spectrum Disorders* | 1764 | 1777 | 2840 | 1581 | 1303 | 1259 | 2043 | 1508 | 1841 | 2783 | 2160 |
| F90-F98 Childhood Behavioral/Emotional Disorders | 29285 | 27374 | 24980 | 23473 | 20033 | 18799 | 19128 | 17487 | 16318 | 15866 | 15158 |
| *F90 ADHD* | 6455 | 5820 | 5553 | 5309 | 5971 | 4483 | 5596 | 4986 | 4744 | 5632 | 5363 |
| *F91-F92 Conduct/Oppositional Disorders* | 11921 | 12058 | 10594 | 10129 | 7263 | 6430 | 7242 | 5417 | 4933 | 3698 | 2698 |
| *F95 Tic Disorders* | 693 | 927 | 584 | 581 | 473 | 328 | 226 | 369 | 427 | 296 | 115 |
| F99-F99 Unspecified Mental Disorder | - | - | 127 | - | - | - | - | - | 52 | 125 | - |
| Diagnosis missing | - | - | - | 26 | - | - | - | - | - | - | - |
| Other Diagnosis | 786 | 1081 | 1421 | 1607 | 1214 | 1480 | 839 | 464 | 420 | 615 | 606 |
| **Adolescent Psychiatry** |  |  |  |  |  |  |  |  |  |  |  |
| *Total inpatient bed days* | 94973 | 85025 | 78834 | 78978 | 86892 | 76408 | 79394 | 73500 | 76875 | 77958 | 83885 |
| F00-F09 Dementia and other organic brain syndromes | 0 | 140 | 78 | - | 8 | 15 | 41 | - | - | - | - |
| F10-F19 Substance Use Disorders | 692 | 598 | 806 | 302 | 556 | 501 | 498 | 725 | 455 | 296 | 290 |
| F20-F29 Schizophrenia-Spectrum Disorders | 16333 | 15748 | 13613 | 14157 | 11389 | 9909 | 11569 | 8896 | 6588 | 7226 | 10169 |
| F30-F39 Mood Disorders | 30738 | 26249 | 22548 | 24272 | 26175 | 23633 | 25685 | 22563 | 23520 | 24789 | 26643 |
| *F30-F31 Mania/Bipolar Disorder* | 3029 | 1992 | 1335 | 2858 | 2343 | 1352 | 1163 | 1062 | 1329 | 990 | 944 |
| *F32-F33 Depressive Disorders* | 26691 | 23673 | 20683 | 20809 | 23006 | 21342 | 23068 | 20224 | 19416 | 21721 | 22819 |
| F40-F48 Neurotic/Stress Disorders | 12815 | 11848 | 13028 | 12191 | 12557 | 11159 | 11085 | 9321 | 10205 | 8529 | 9581 |
| *F40-F41 Anxiety Disorders* | 6576 | 7210 | 7649 | 6876 | 7449 | 7379 | 7425 | 6473 | 6870 | 6034 | 6561 |
| *F42 OCD* | 2717 | 2256 | 1405 | 1648 | 1954 | 1187 | 1048 | 1299 | 2207 | 1129 | 1471 |
| F50-F59 Behavioural physiological/physical factors | 12300 | 11636 | 9958 | 10014 | 13598 | 10632 | 11969 | 12855 | 15503 | 16650 | 18256 |
| *F50 Eating Disorders* | 12210 | 11543 | 9851 | 9840 | 13473 | 10530 | 11944 | 12845 | 15461 | 16566 | 18225 |
| F60-F69 Personality Disorders | 456 | 60 | 474 | 445 | 872 | 1391 | 809 | 786 | 696 | 430 | 437 |
| *F60-F61 Adult personality/behavioral Disorders* | 338 | - | 198 | 242 | 522 | 1031 | 475 | 595 | 680 | 410 | 343 |
| *F64 Gender Identity Disorders* | - | - | - | - | - | - | - | - | - | - | - |
| F70-F79 Intellectual Disabilities | 239 | 265 | 487 | 631 | 446 | 529 | 998 | 882 | 747 | 772 | 882 |
| F80-F89 Psychological Development Disorders | 2563 | 2471 | 4024 | 3587 | 4290 | 4039 | 3314 | 3495 | 3373 | 4873 | 3311 |
| *F84.0-F84.1,F84.5 Autism Spectrum Disorders* | 1332 | 1129 | 1979 | 1460 | 2760 | 2523 | 1745 | 1768 | 2072 | 2707 | 1475 |
| F90-F98 Childhood Behavioral/Emotional Disorders | 17670 | 15009 | 12080 | 11195 | 15185 | 12431 | 11322 | 11932 | 13494 | 11145 | 12149 |
| *F90 ADHD* | 2598 | 1784 | 2709 | 1350 | 3330 | 1932 | 2208 | 1752 | 2159 | 1859 | 1810 |
| *F91-F92 Conduct/Oppositional Disorders* | 7402 | 5592 | 4314 | 4474 | 6538 | 4229 | 3325 | 4224 | 3564 | 1989 | 2209 |
| *F95 Tic Disorders* | 173 | 290 | 253 | 381 | 162 | 551 | 302 | 56 | 247 | 88 | 112 |
| F99-F99 Unspecified Mental Disorder | 76 | 5 | 202 | 54 | 130 | 231 | 258 | 10 | 174 | 582 | 54 |
| Diagnosis missing | 512 | - | 206 | 245 | - | - | - | - | - | - | - |
| Other Diagnosis | 579 | 996 | 1330 | 1885 | 1686 | 1938 | 1846 | 2035 | 2120 | 2666 | 2113 |

*Note.* Blank cells indicate that there were too few observations to report (<5) or that data was not available

Supplementary Table 3. Numbers of individuals with inpatient psychiatry admissions for children and adolescents in Finland, 2013-2023

|  | **Number of Individuals with an Inpatient Admission** | | | | | | | | | | |
| --- | --- | --- | --- | --- | --- | --- | --- | --- | --- | --- | --- |
| **Child Psychiatry** | **2013** | **2014** | **2015** | **2016** | **2017** | **2018** | **2019** | **2020** | **2021** | **2022** | **2023** |
| *Total individuals with inpatient admission* | 1069 | 1059 | 1139 | 1096 | 1163 | 1139 | 1140 | 1039 | 1103 | 1042 | 1164 |
| F00-F09 Dementia and other organic brain syndromes | - | - | - | - | - | - | - | - | - | - | - |
| F10-F19 Substance Use Disorders | - | - | - | - | - | - | - | - | - | - | - |
| F20-F29 Schizophrenia-Spectrum Disorders | 22 | 22 | 16 | 14 | 20 | 15 | 11 | 14 | 17 | 13 | 16 |
| F30-F39 Mood Disorders | 138 | 106 | 122 | 137 | 162 | 163 | 172 | 129 | 168 | 144 | 185 |
| *F30-F31 Mania/Bipolar Disorder* | 14 | 9 | 6 | 5 | - | 6 | 5 | - | - | - | - |
| *F32-F33 Depressive Disorders* | 114 | 86 | 103 | 116 | 130 | 135 | 133 | 97 | 122 | 112 | 142 |
| F40-F48 Neurotic/Stress Disorders | 99 | 112 | 113 | 98 | 124 | 137 | 99 | 125 | 134 | 115 | 128 |
| *F40-F41 Anxiety Disorders* | 29 | 40 | 24 | 16 | 40 | 75 | 48 | 52 | 64 | 61 | 73 |
| *F42 OCD* | 13 | 16 | 17 | 23 | 19 | 15 | 11 | 16 | 18 | 15 | 18 |
| F50-F59 Behavioural physiological/physical factors | 18 | 24 | 26 | 22 | 20 | 32 | 40 | 40 | 46 | 59 | 52 |
| *F50 Eating Disorders* | 17 | 23 | 25 | 20 | 19 | 31 | 40 | 40 | 46 | 59 | 51 |
| F60-F69 Personality Disorders | - | - | - | - | - | - | - | - | - | - | - |
| *F60-F61 Adult personality/behavioral Disorders* | - | - | - | - | - | - | - | - | - | - | - |
| *F64 Gender Identity Disorders* | - | - | - | - | - | - | - | - | - | - | - |
| F70-F79 Intellectual Disabilities | 10 | 11 | 8 | 10 | 12 | 8 | 6 | 9 | 8 | 5 | 5 |
| F80-F89 Psychological Development Disorders | 130 | 127 | 165 | 144 | 153 | 111 | 143 | 122 | 114 | 125 | 128 |
| *F84.0-F84.1,F84.5 Autism Spectrum Disorders* | 38 | 40 | 61 | 39 | 36 | 38 | 62 | 48 | 54 | 66 | 63 |
| F90-F98 Childhood Behavioral/Emotional Disorders | 609 | 600 | 608 | 599 | 619 | 622 | 618 | 564 | 579 | 534 | 608 |
| *F90 ADHD* | 126 | 134 | 138 | 128 | 177 | 155 | 164 | 170 | 172 | 188 | 223 |
| *F91-F92 Conduct/Oppositional Disorders* | 288 | 267 | 268 | 283 | 237 | 237 | 239 | 176 | 180 | 140 | 147 |
| *F95 Tic Disorders* | 11 | 20 | 15 | 12 | 16 | 14 | - | 14 | 14 | 8 | 8 |
| F99-F99 Unspecified Mental Disorder | - | - | - | - | - | - | - | - | - | - | - |
| Diagnosis missing | - | - | - | - | - | - | - | - | - | - | - |
| Other Diagnosis | 43 | 57 | 81 | 64 | 53 | 51 | 51 | 36 | 37 | 47 | 42 |
| **Adolescent Psychiatry** |  |  |  |  |  |  |  |  |  |  |  |
| *Total individuals with inpatient admission* | 2384 | 2315 | 2263 | 2508 | 3006 | 3035 | 3201 | 3354 | 3605 | 3447 | 3536 |
| F00-F09 Dementia and other organic brain syndromes | - | - | - | - | - | - | - | - | - | - | - |
| F10-F19 Substance Use Disorders | 47 | 49 | 46 | 41 | 74 | 87 | 86 | 149 | 82 | 67 | 64 |
| F20-F29 Schizophrenia-Spectrum Disorders | 174 | 195 | 177 | 198 | 165 | 164 | 169 | 156 | 138 | 136 | 147 |
| F30-F39 Mood Disorders | 819 | 795 | 758 | 891 | 1123 | 1124 | 1244 | 1195 | 1386 | 1285 | 1376 |
| *F30-F31 Mania/Bipolar Disorder* | 70 | 46 | 42 | 52 | 59 | 56 | 42 | 42 | 52 | 45 | 40 |
| *F32-F33 Depressive Disorders* | 723 | 732 | 702 | 823 | 1041 | 1022 | 1107 | 1040 | 1213 | 1156 | 1185 |
| F40-F48 Neurotic/Stress Disorders | 444 | 427 | 474 | 505 | 587 | 558 | 595 | 622 | 659 | 598 | 618 |
| *F40-F41 Anxiety Disorders* | 262 | 269 | 302 | 331 | 398 | 388 | 459 | 485 | 494 | 474 | 477 |
| *F42 OCD* | 42 | 41 | 38 | 29 | 37 | 38 | 34 | 48 | 72 | 54 | 58 |
| F50-F59 Behavioural physiological/physical factors | 151 | 168 | 136 | 137 | 183 | 181 | 191 | 243 | 274 | 327 | 296 |
| *F50 Eating Disorders* | 148 | 162 | 125 | 129 | 176 | 176 | 186 | 240 | 271 | 324 | 293 |
| F60-F69 Personality Disorders | 13 | 5 | 13 | 14 | 25 | 35 | 26 | 33 | 27 | 18 | 26 |
| *F60-F61 Adult personality/behavioral Disorders* | 9 | - | 9 | 10 | 19 | 29 | 21 | 29 | 24 | 16 | 20 |
| *F64 Gender Identity Disorders* | - | - | - | - | - | - | - | - | - | - | - |
| F70-F79 Intellectual Disabilities | 11 | 16 | 24 | 22 | 28 | 25 | 28 | 39 | 28 | 34 | 38 |
| F80-F89 Psychological Development Disorders | 84 | 84 | 95 | 110 | 122 | 130 | 121 | 130 | 151 | 162 | 154 |
| *F84.0-F84.1,F84.5 Autism Spectrum Disorders* | 44 | 41 | 41 | 47 | 75 | 76 | 55 | 68 | 86 | 86 | 85 |
| F90-F98 Childhood Behavioral/Emotional Disorders | 576 | 527 | 444 | 489 | 577 | 595 | 597 | 624 | 664 | 645 | 653 |
| *F90 ADHD* | 69 | 63 | 64 | 56 | 109 | 111 | 120 | 115 | 120 | 120 | 120 |
| *F91-F92 Conduct/Oppositional Disorders* | 261 | 246 | 200 | 208 | 240 | 207 | 195 | 217 | 169 | 144 | 146 |
| *F95 Tic Disorders* | 7 | 8 | 11 | 7 | - | 10 | 16 | 6 | 6 | 6 | 9 |
| F99-F99 Unspecified Mental Disorder | - | - | - | 7 | 9 | 10 | 7 | - | 11 | 6 | - |
| Diagnosis missing | 10 | 0 | 7 | 8 | - | - | - | - | - | - | - |
| Other Diagnosis | 55 | 49 | 89 | 86 | 113 | 126 | 137 | 163 | 185 | 169 | 164 |

*Note.* Blank cells indicate that there were too few observations to report (<5)

Supplementary Table 4. Numbers of outpatient psychiatry contacts (including remote contacts) for children and adolescents in Finland, 2013-2023

|  | **Number of Outpatient Psychiatry Contacts** | | | | | | | | | | |
| --- | --- | --- | --- | --- | --- | --- | --- | --- | --- | --- | --- |
| **Child Psychiatry** | **2013** | **2014** | **2015** | **2016** | **2017** | **2018** | **2019** | **2020** | **2021** | **2022** | **2023** |
| *Total Outpatient contacts* | 165214 | 177880 | 204902 | 220920 | 237628 | 246420 | 239857 | 242332 | 255874 | 251736 | 268215 |
| F00-F09 Dementia and other organic brain syndromes | 14 | 24 | 5 | 22 | 19 | 47 | 22 | 24 | 25 | 6 | - |
| F10-F19 Substance Use Disorders | 6 | 18 | 8 | 5 | 16 | 6 | 8 | 7 | 18 | - | 5 |
| F20-F29 Schizophrenia-Spectrum Disorders | 548 | 502 | 553 | 750 | 807 | 552 | 487 | 568 | 568 | 699 | 565 |
| F30-F39 Mood Disorders | 6020 | 7480 | 8071 | 9065 | 9580 | 9443 | 9586 | 9560 | 10249 | 8467 | 8537 |
| *F30-F31 Mania/Bipolar Disorder* | 443 | 409 | 317 | 152 | 262 | 203 | 131 | 214 | 198 | 113 | 119 |
| *F32-F33 Depressive Disorders* | 5221 | 6055 | 6503 | 7294 | 7973 | 8054 | 8113 | 7896 | 8843 | 7527 | 7145 |
| F40-F48 Neurotic/Stress Disorders | 9933 | 12032 | 13635 | 15423 | 16901 | 17662 | 17734 | 18522 | 19675 | 19798 | 21609 |
| *F40-F41 Anxiety Disorders* | 2623 | 3528 | 3834 | 4037 | 5384 | 6535 | 6108 | 6905 | 9233 | 8705 | 10586 |
| *F42 OCD* | 1554 | 2404 | 2427 | 2856 | 2694 | 3161 | 3472 | 3849 | 3612 | 3785 | 4048 |
| F50-F59 Behavioural physiological/physical factors | 2610 | 2805 | 3181 | 2879 | 2649 | 2971 | 2883 | 3335 | 3726 | 4138 | 4054 |
| *F50 Eating Disorders* | 1365 | 1633 | 1978 | 1719 | 1778 | 1986 | 1930 | 2492 | 2897 | 3566 | 3626 |
| F60-F69 Personality Disorders | 131 | 139 | 133 | 195 | 204 | 228 | 204 | 249 | 177 | 229 | 118 |
| *F60-F61 Adult personality/behavioral Disorders* | - | - | - | - | - | - | - | - | - | 5 | - |
| *F64 Gender Identity Disorders* | - | - | - | - | 11 | 10 | 16 | 19 | 30 | 27 | - |
| F70-F79 Intellectual Disabilities | 232 | 322 | 339 | 447 | 393 | 337 | 535 | 527 | 437 | 484 | 433 |
| F80-F89 Psychological Development Disorders | 16446 | 20100 | 25568 | 30052 | 35321 | 36739 | 37699 | 36069 | 35533 | 34497 | 38997 |
| *F84.0-F84.1,F84.5 Autism Spectrum Disorders* | 5405 | 6628 | 7466 | 6722 | 7834 | 8828 | 9451 | 10849 | 10661 | 11109 | 12855 |
| F90-F98 Childhood Behavioral/Emotional Disorders | 72322 | 88217 | 98984 | 104012 | 113212 | 117012 | 115356 | 122557 | 136459 | 139126 | 147628 |
| *F90 ADHD* | 18655 | 24973 | 30920 | 35007 | 39728 | 43674 | 44906 | 53679 | 64349 | 70901 | 77793 |
| *F91-F92 Conduct/Oppositional Disorders* | 19965 | 23647 | 24845 | 23501 | 24784 | 22908 | 21459 | 21365 | 19718 | 17435 | 16069 |
| *F95 Tic Disorders* | 1464 | 1861 | 2227 | 2642 | 2502 | 2479 | 2244 | 2510 | 3272 | 3141 | 3657 |
| F99-F99 Unspecified Mental Disorder | 53 | 52 | 28 | 33 | 10 | 29 | 53 | 36 | 76 | 95 | 84 |
| Diagnosis missing | 25643 | 16541 | 19843 | 18450 | 11134 | 12577 | 14166 | 17145 | 15651 | 12451 | 14145 |
| Other Diagnosis | 31256 | 29648 | 34554 | 39587 | 47382 | 48817 | 41124 | 33733 | 33280 | 31746 | 32040 |
| **Adolescent Psychiatry** |  |  |  |  |  |  |  |  |  |  |  |
| *Total Outpatient contacts* | 234298 | 246360 | 263486 | 275424 | 317309 | 322771 | 315609 | 331311 | 364043 | 345906 | 352047 |
| F00-F09 Dementia and other organic brain syndromes | 26 | 36 | 17 | 18 | 19 | 24 | 11 | 7 | 6 | 26 | 39 |
| F10-F19 Substance Use Disorders | 890 | 1350 | 1103 | 933 | 876 | 1342 | 1431 | 1321 | 760 | 707 | 1064 |
| F20-F29 Schizophrenia-Spectrum Disorders | 5618 | 6923 | 6825 | 6542 | 6012 | 4445 | 5006 | 5129 | 4600 | 4818 | 4782 |
| F30-F39 Mood Disorders | 52903 | 60746 | 62988 | 69879 | 90366 | 95157 | 89501 | 91827 | 101115 | 95430 | 90654 |
| *F30-F31 Mania/Bipolar Disorder* | 2262 | 2420 | 1996 | 1852 | 2703 | 2460 | 1932 | 1897 | 2244 | 2255 | 2318 |
| *F32-F33 Depressive Disorders* | 48681 | 56334 | 58711 | 65069 | 83954 | 88960 | 83800 | 84750 | 90190 | 81969 | 78894 |
| F40-F48 Neurotic/Stress Disorders | 44499 | 53653 | 58604 | 63376 | 71955 | 69697 | 68584 | 75546 | 80006 | 74181 | 75121 |
| *F40-F41 Anxiety Disorders* | 28105 | 35639 | 40535 | 44920 | 51081 | 50417 | 48909 | 55578 | 59717 | 55288 | 56132 |
| *F42 OCD* | 4732 | 5643 | 6475 | 6657 | 7111 | 6905 | 7149 | 8272 | 9800 | 9741 | 9988 |
| F50-F59 Behavioural physiological/physical factors | 16405 | 17246 | 15999 | 15253 | 16846 | 18993 | 20041 | 21475 | 26627 | 26069 | 25604 |
| *F50 Eating Disorders* | 15667 | 16679 | 15190 | 14202 | 15911 | 18080 | 19248 | 21092 | 26327 | 25856 | 25376 |
| F60-F69 Personality Disorders | 998 | 1257 | 1534 | 1799 | 2022 | 1908 | 1604 | 2232 | 1917 | 1512 | 1270 |
| *F60-F61 Adult personality/behavioral Disorders* | 208 | 263 | 357 | 340 | 671 | 675 | 591 | 941 | 1030 | 547 | 486 |
| *F64 Gender Identity Disorders* | 616 | 793 | 947 | 1305 | 1168 | 1003 | 869 | 1208 | 817 | 840 | 670 |
| F70-F79 Intellectual Disabilities | 247 | 373 | 500 | 660 | 660 | 626 | 534 | 660 | 753 | 531 | 464 |
| F80-F89 Psychological Development Disorders | 10231 | 11554 | 13212 | 12949 | 16033 | 16081 | 16924 | 16827 | 18984 | 18976 | 22110 |
| *F84.0-F84.1,F84.5 Autism Spectrum Disorders* | 6115 | 6630 | 6791 | 6470 | 8597 | 8417 | 8484 | 9541 | 10298 | 10143 | 11621 |
| F90-F98 Childhood Behavioral/Emotional Disorders | 39627 | 41871 | 43919 | 45503 | 48693 | 51139 | 51662 | 54461 | 63467 | 65667 | 71348 |
| *F90 ADHD* | 9391 | 10621 | 11969 | 13381 | 14593 | 16351 | 18119 | 21933 | 26232 | 29578 | 36416 |
| *F91-F92 Conduct/Oppositional Disorders* | 12631 | 12245 | 11330 | 10930 | 11519 | 11107 | 10192 | 8703 | 7695 | 6116 | 6083 |
| *F95 Tic Disorders* | 753 | 795 | 901 | 976 | 1071 | 1150 | 1185 | 1500 | 2145 | 1695 | 1421 |
| F99-F99 Unspecified Mental Disorder | 288 | 750 | 721 | 331 | 530 | 1090 | 876 | 456 | 294 | 223 | 163 |
| Diagnosis missing | 35715 | 22128 | 28204 | 24883 | 21755 | 20861 | 21473 | 26576 | 29560 | 24371 | 26763 |
| Other Diagnosis | 26851 | 28473 | 29860 | 33298 | 41542 | 41408 | 37962 | 34794 | 35954 | 33395 | 32665 |

*Note.* Blank cells indicate that there were too few observations to report (<5)

Supplementary Table 5. Numbers of individuals with outpatient psychiatry contacts (including remote contacts) for children and adolescents in Finland, 2013-2023

|  | **Number of Individuals with an Outpatient Psychiatry Contact** | | | | | | | | | | |
| --- | --- | --- | --- | --- | --- | --- | --- | --- | --- | --- | --- |
| **Child Psychiatry** | **2013** | **2014** | **2015** | **2016** | **2017** | **2018** | **2019** | **2020** | **2021** | **2022** | **2023** |
| *Total individuals with outpatient contacts* | 21191 | 23211 | 22404 | 25423 | 27950 | 29720 | 36110 | 41821 | 48761 | 47227 | 50329 |
| F00-F09 Dementia and other organic brain syndromes | - | 6 | - | - | - | - | - | - | - | - | - |
| F10-F19 Substance Use Disorders | - | 5 | 5 | - | - | 5 | 5 | - | 5 | - | - |
| F20-F29 Schizophrenia-Spectrum Disorders | 64 | 60 | 48 | 53 | 65 | 46 | 66 | 73 | 70 | 77 | 53 |
| F30-F39 Mood Disorders | 784 | 841 | 772 | 802 | 827 | 906 | 1092 | 1287 | 1505 | 1305 | 1260 |
| *F30-F31 Mania/Bipolar Disorder* | 48 | 42 | 24 | 13 | 11 | 19 | 17 | 20 | 29 | 21 | 15 |
| *F32-F33 Depressive Disorders* | 689 | 718 | 600 | 666 | 722 | 798 | 985 | 1087 | 1283 | 1118 | 1033 |
| F40-F48 Neurotic/Stress Disorders | 1287 | 1453 | 1392 | 1514 | 1698 | 1693 | 2271 | 2733 | 3120 | 3050 | 3089 |
| *F40-F41 Anxiety Disorders* | 382 | 456 | 409 | 451 | 562 | 640 | 824 | 985 | 1385 | 1376 | 1519 |
| *F42 OCD* | 179 | 238 | 211 | 214 | 247 | 240 | 392 | 498 | 566 | 581 | 607 |
| F50-F59 Behavioural physiological/physical factors | 441 | 436 | 350 | 374 | 311 | 318 | 455 | 573 | 622 | 642 | 591 |
| *F50 Eating Disorders* | 125 | 165 | 149 | 148 | 149 | 163 | 207 | 298 | 358 | 471 | 458 |
| F60-F69 Personality Disorders | 35 | 34 | 25 | 29 | 26 | 27 | 34 | 50 | 47 | 44 | 28 |
| *F60-F61 Adult personality/behavioral Disorders* | - | - | - | - | - | - | - | - | - | - | - |
| *F64 Gender Identity Disorders* | - | - | - | - | - | - | 7 | 6 | 10 | 5 | - |
| F70-F79 Intellectual Disabilities | 72 | 90 | 84 | 98 | 108 | 90 | 130 | 148 | 136 | 148 | 121 |
| F80-F89 Psychological Development Disorders | 2079 | 2595 | 2685 | 3098 | 3540 | 3869 | 5466 | 6580 | 7471 | 7404 | 8195 |
| *F84.0-F84.1,F84.5 Autism Spectrum Disorders* | 673 | 829 | 805 | 819 | 909 | 1036 | 1427 | 1950 | 2438 | 2465 | 2548 |
| F90-F98 Childhood Behavioral/Emotional Disorders | 7615 | 8756 | 8636 | 9388 | 10394 | 11076 | 14938 | 18127 | 22026 | 21940 | 22684 |
| *F90 ADHD* | 2647 | 3248 | 3085 | 3561 | 4139 | 4682 | 6778 | 9321 | 12191 | 12738 | 13656 |
| *F91-F92 Conduct/Oppositional Disorders* | 2466 | 2715 | 2350 | 2311 | 2312 | 2214 | 2686 | 3210 | 3466 | 3125 | 2740 |
| *F95 Tic Disorders* | 224 | 264 | 227 | 275 | 283 | 301 | 369 | 547 | 810 | 795 | 835 |
| F99-F99 Unspecified Mental Disorder | 13 | 21 | 9 | 11 | - | 8 | 14 | 14 | 17 | 18 | 13 |
| Diagnosis missing | 2903 | 2680 | 2380 | 2970 | 2489 | 3036 | 2677 | 3582 | 4096 | 3736 | 5206 |
| Other Diagnosis | 5898 | 6234 | 6018 | 7086 | 8492 | 8646 | 8962 | 8654 | 9646 | 8863 | 9089 |
| **Adolescent Psychiatry** |  |  |  |  |  |  |  |  |  |  |  |
| *Total individuals with outpatient contacts* | 29693 | 31863 | 29334 | 31929 | 36228 | 37670 | 42676 | 53081 | 61333 | 61353 | 63079 |
| F00-F09 Dementia and other organic brain syndromes | 7 | 8 | 7 | 5 | 12 | 8 | 6 | - | - | - | - |
| F10-F19 Substance Use Disorders | 220 | 222 | 221 | 182 | 210 | 291 | 309 | 401 | 262 | 245 | 279 |
| F20-F29 Schizophrenia-Spectrum Disorders | 529 | 512 | 442 | 459 | 475 | 374 | 468 | 558 | 579 | 595 | 544 |
| F30-F39 Mood Disorders | 5120 | 5553 | 5039 | 5648 | 6800 | 7328 | 8711 | 11040 | 12943 | 12800 | 11815 |
| *F30-F31 Mania/Bipolar Disorder* | 225 | 209 | 184 | 160 | 190 | 186 | 210 | 235 | 287 | 312 | 305 |
| *F32-F33 Depressive Disorders* | 4819 | 5186 | 4695 | 5297 | 6362 | 6908 | 8192 | 10233 | 11667 | 11248 | 10507 |
| F40-F48 Neurotic/Stress Disorders | 5123 | 5916 | 5545 | 5948 | 6713 | 6654 | 8124 | 10813 | 12415 | 12337 | 12204 |
| *F40-F41 Anxiety Disorders* | 3333 | 4029 | 3795 | 4139 | 4793 | 4862 | 5950 | 8104 | 9610 | 9623 | 9530 |
| *F42 OCD* | 505 | 576 | 535 | 589 | 628 | 630 | 840 | 1195 | 1504 | 1643 | 1625 |
| F50-F59 Behavioural physiological/physical factors | 1276 | 1305 | 1162 | 1120 | 1168 | 1266 | 1611 | 2109 | 2598 | 2840 | 2707 |
| *F50 Eating Disorders* | 1096 | 1170 | 1007 | 933 | 1006 | 1112 | 1433 | 1951 | 2470 | 2727 | 2599 |
| F60-F69 Personality Disorders | 145 | 166 | 190 | 283 | 334 | 321 | 364 | 525 | 401 | 364 | 321 |
| *F60-F61 Adult personality/behavioral Disorders* | 34 | 37 | 33 | 49 | 58 | 60 | 68 | 96 | 120 | 102 | 73 |
| *F64 Gender Identity Disorders* | 80 | 95 | 124 | 211 | 245 | 226 | 259 | 397 | 251 | 233 | 206 |
| F70-F79 Intellectual Disabilities | 94 | 117 | 108 | 128 | 119 | 144 | 153 | 174 | 198 | 187 | 183 |
| F80-F89 Psychological Development Disorders | 1404 | 1663 | 1676 | 1752 | 1946 | 2084 | 2750 | 3455 | 4301 | 4757 | 5334 |
| *F84.0-F84.1,F84.5 Autism Spectrum Disorders* | 756 | 860 | 842 | 819 | 935 | 1024 | 1371 | 1769 | 2224 | 2470 | 2715 |
| F90-F98 Childhood Behavioral/Emotional Disorders | 5419 | 5892 | 5299 | 5679 | 6093 | 6386 | 8091 | 10173 | 12375 | 13178 | 14103 |
| *F90 ADHD* | 1667 | 1911 | 1724 | 1953 | 2214 | 2520 | 3480 | 4840 | 6376 | 7272 | 8426 |
| *F91-F92 Conduct/Oppositional Disorders* | 1840 | 1772 | 1519 | 1497 | 1436 | 1409 | 1648 | 1850 | 1644 | 1472 | 1271 |
| *F95 Tic Disorders* | 122 | 139 | 127 | 136 | 150 | 147 | 196 | 315 | 460 | 430 | 377 |
| F99-F99 Unspecified Mental Disorder | 144 | 300 | 163 | 70 | 120 | 183 | 168 | 114 | 91 | 69 | 68 |
| Diagnosis missing | 4255 | 3929 | 3760 | 3891 | 4013 | 4664 | 3991 | 4698 | 5574 | 5070 | 7025 |
| Other Diagnosis | 5957 | 6280 | 5722 | 6764 | 8225 | 7967 | 7930 | 9021 | 9596 | 8911 | 8496 |

*Note.* Blank cells indicate that there were too few observations to report (<5)
